# Computation of Vascular Parameters: Implementing Methodology and Performance Analysis

**DOI:** 10.3390/bios13080757

**Published:** 2023-07-25

**Authors:** Mohamed Yacin Sikkandar, Sridharan Padmanabhan, Bobby Mohan, Ibrahim AlMohimeed, Ahmad Alassaf, Shady A. Alshewaier, Ali Abdullah Almukil, Sabarunisha Begum

**Affiliations:** 1 Department of Medical Equipment Technology, College of Applied Medical Sciences, Majmaah University, Al Majmaah 11952, Saudi Arabia; 2 Department of Biomedical Engineering, Rajalakshmi Engineering College, Chennai 602105, India; 3 Department of Physical Therapy, College of Applied Medical Sciences, Majmaah University, Al Majmaah 11952, Saudi Arabia; 4 Department of Biotechnology, P.S.R. Engineering College, Sivakasi 626140, India

**Keywords:** arterial compliance, stiffness index, pulse wave velocity, blood pressure, acoustic waves

## Abstract

This paper presents the feasibility of automated and accurate in vivo measurements of vascular parameters using an ultrasound sensor. The continuous and non-invasive monitoring of certain parameters, such as pulse wave velocity (PWV), blood pressure (BP), arterial compliance (AC), and stiffness index (SI), is crucial for assessing cardiovascular disorders during surgeries and follow-up procedures. Traditional methods, including cuff-based or invasive catheter techniques, serve as the gold standard for measuring BP, which is then manually used to calculate AC and SI through imaging algorithms. In this context, the Continuous and Non-Invasive Vascular Stiffness and Arterial Compliance Screener (CaNVAS) is developed to provide continuous and non-invasive measurements of these parameters using an ultrasound sensor. By driving 5 MHz (ranging from 2.2 to 10 MHz) acoustic waves through the arterial walls, capturing the reflected echoes, and employing pre-processing techniques, the frequency shift is utilized to calculate PWV. It is observed that PWV measured by CaNVAS correlates exponentially with BP values obtained from the sphygmomanometer (BPMR-120), enabling the computation of instantaneous BP values. The proposed device is validated through measurements conducted on 250 subjects under pre- and post-exercise conditions, demonstrating an accuracy of 95% and an average coefficient of variation of 12.5%. This validates the reliability and precision of CaNVAS in assessing vascular parameters.

## 1. Introduction

According to the World Health Organization (WHO), cardiovascular diseases (CVD) are the leading cause of global mortality, affecting approximately 17.9 million individuals annually. Hypertension has impacted an estimated 1.13 billion people worldwide, with the majority residing in low- and middle-income countries. Those with hypertension are at a higher risk of developing severe medical complications, including brain, heart, and kidney diseases, among others. Disturbingly, reports indicate that 1 in 4 men and 1 in 5 women suffer from hypertension, making it a significant contributor to premature mortality on a global scale. Physiologically, pulse wave velocity (PWV), blood pressure (BP), arterial compliance (AC), and stiffness index (SI) are closely related vascular parameters and play an important role in maintaining blood flow to all vital organs [1,2,3]. Studies have demonstrated a significant correlation between these four parameters and cardiovascular diseases [4,5,6]. Measuring and monitoring these parameters non-invasively and continuously is a main concern for clinicians, and there is a need for an automated device, specifically throughout prolonged medical procedures like dialysis and surgeries. The first conference of consensus on arterial stiffness (AS) held in June 2000 (Paris, France) summarized the existing devices for measuring arterial compliance depending on principles like pulse transmit time, arterial pressure pulse, and vessel diameter, but these are targeted only to measure arterial compliance [7]. Manual measurements of blood pressure based on the gold standard sphygmomanometer demands focused attention and can lead to human errors if operated by an unskilled individual. A non-invasive measurement of AS and AC using traditional oscillometric blood pressure measurement was carried out by Hidehiko et al. [8]. A calibration-free Photoplethysmography (PPG) waveform analysis and a biometric-based technique were used to measure BP [9]. In addition to the above theoretical procedures, there are many patented devices available to monitor BP non-invasively and continuously using cuff-based pressure sensors [10,11,12,13]. Hoctor et al. developed an ultrasonic-based method and apparatus to measure BP through a continuous and non-invasive method by capturing images using ultrasound to derive blood pressure values [14]. Recently, Yamil Kuri developed a system and method to determine AC and SI. This system was configured to calculate BP based on calculating the blood flow velocity and subsequently derived AC and SI [15,16]. Polanczyk A et al. have conducted hemodynamic studies on a three-dimensional reconstruction of the blood vessel and describe its biomechanical properties [17,18,19].

All these devices monitor PWV, BP, AC, and SI parameters individually or collectively, either by causing discomfort to the subject with continuous measurements or image-guided ultrasound modalities with complex algorithms. Also, these devices require a large set-up for monitoring in intensive clinical environments, which makes it more complex for implementation in basic healthcare units in remote areas. Computer- and image-assisted methods of diagnosis have high accuracy, but repeatability remains a big area to be worked on. Many researchers have discussed carotid stiffness indices, which depend on the relationship between the pressure and the diameter of arterial distension from the diastolic to the systolic phase, giving deep insight into the relationship between geometrical parameters of the blood vessel and clinical values [13,20,21,22,23,24,25,26,27,28,29]. The in vivo non-invasive technique that employed Young’s modulus estimated from the regional stress–strain relationship conveyed wisdom learning on arterial wall properties [30]. Few studies have worked on the in vivo Young’s modulus measurement of the pressure–strain relationship on the carotid artery or based on the slope of the stress–strain relationship at end-diastole and end-systole. Young’s modulus of the arteries, which is a relationship between blood pressure and arterial diameter, is found to indicate profound variations for different individuals [31,32,33,34,35,36]. Existing ultrasound imaging has been widely employed in the imaging of soft tissues, specifically blood vessels, for diagnosing cardiovascular disorders. The movement of the arterial wall can be measured using the M-Mode ultrasound method of estimation or using RF signals. These imaging methods have their targets specifically on brachial, femoral, and carotid arteries and abdominal aorta [37]. Furthermore, 1D cross-correlation methods have been employed to estimate carotid distention waveform and to visualize its structure [38].

A survey of the literature indicates that existing modalities employed to find blood pressure and arterial parameters have many impediments. Frequent usage of cuff-based devices may lead to damage of the blood vessels, and a skilled technician is required to detect the Korotkoff sound to evaluate BP. Employing separate modalities like imaging algorithms for finding arterial parameters and invasive catheter methods to detect continuous blood pressure leads to an increase in complexity and a potential clinical hazard that is unavoidable during long-term surgical procedures.

Clustering all of these important parameters into a single continuous non-invasive device without a complex imaging algorithm would benefit clinical specialists to successfully carry out prolonged and lifesaving surgeries. In this context, the aim of this research is to develop a device that will measure said parameters continuously and non-invasively using an ultrasound sensor. This paper provides additional confirmation of the potential clinical application of a Continuous and Non-Invasive Vascular Stiffness and Arterial Compliance Screener (CaNVAS) device by demonstrating the feasibility of automated and precise in vivo measurements of these parameters using a straightforward and innovative methodology. This validation paves the way for the device’s potential use in clinical settings in the near future.

## 2. Materials and Methods

This device focuses on deriving the blood pressure value from PWV; in addition, AC and SI are computed, making it a suitable alternative for existing modalities. Nonionizing ultrasound waves are passed through the blood vessel to obtain the reflected echoes to evaluate the PWV using the Doppler shift. The relationship between PWV and BP is established, which helps in the automated estimation of BP from PWV. AC and SI are formulated by extracting the derived systolic and diastolic diameter and blood pressure. To validate the entire experimental set-up in its working, two checkpoints with the artery diameter value as an indicator are programmed to indicate any deviation on device placement in the region of interest. A vessel finder is utilized to trace the artery position or to correct the CaNVAS position whenever needed. CaNVAS is developed to monitor vascular parameters under a single roof in a feasible manner without involving any complex algorithm, making it extendable to be employed as a point-of-care device for CVD patients.

### 2.1. CaNVAS: System Architecture

**Figure 1:**
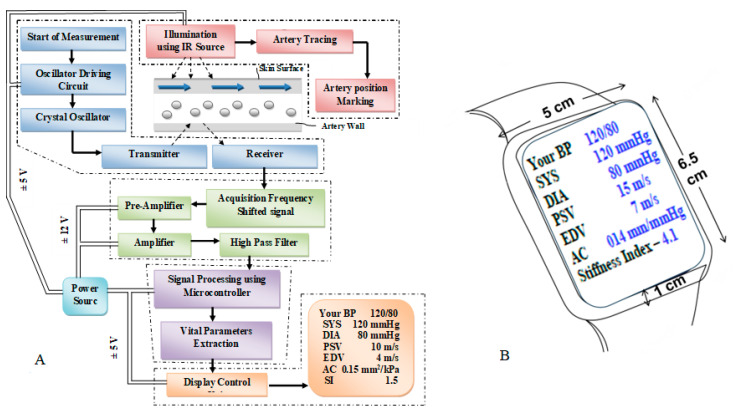
(**A**). CaNVAS system architecture (**B**). CaNVAS device sketch.

Figure 1A illustrates the comprehensive architecture of CaNVAS, which consists of various modules essential for measuring vascular parameters. These modules include:

(i) A sensor positioning module equipped with infrared illumination to mark the artery’s position.
(ii) An embedded ultrasound sensor module housing a piezoelectric crystal oscillator with an appropriate frequency.
(iii) A transmitter unit, receiver unit, and pre-processing module encompassing an acquisition unit, operational amplifier, and high-pass filter.
(iv) A microprocessor and signal processing module responsible for extracting vascular parameters.
(v) A display module featuring a manual setting mode and an output display unit.

It is important to note that the term “vascular parameters” encompasses systolic blood pressure (P_s_), diastolic blood pressure (P_d_), pulse wave velocity (PWV) (which includes peak systolic velocity (V_s_) and end diastolic velocity (V_d_)), arterial compliance (AC), and stiffness index (SI).

#### 2.1.1. Sensor Positioning Module

A sensor positioning module is provided with an artery tracing circuit to support the non-invasive device in marking the correct position of the target artery on the skin’s surface, thereby illuminating the infrared source at a wavelength of 620 nm to visualize the target artery. Figure 1B shows the device sketch in which the sensor is positioned on the surface of the wrist underneath the display panel. The device includes an ultrasound sensor transmitter that is embedded with a piezoelectric crystal oscillator and a driver circuit. This transmitter emits a continuous acoustic wave at a frequency of F_t_. At the other end, the receiver module picks up the reflected acoustic wave F_O_, which exhibits a distinct frequency compared to the transmitted frequency. This frequency difference between the transmitter and receiver modules is known as the Doppler shift (F_S_). The device transmits acoustic waves at a predetermined frequency across the blood vessel interface to capture reflected waves from the vessel’s surface, causing a frequency shift in the receiver module. Both the transmitter and receiver modules are positioned adjacent to each other on the skin’s surface, aligned with the direction of blood flow towards the receiver module. A crystal oscillator embedded in the sensor activates the ultrasonic transmitter module, generating waves at a specific frequency that interact with the blood vessel. After being absorbed, the waves are reflected back and detected through the oscillator crystal in the receiver module. The programmed microprocessor controls the crystal oscillator, producing specific frequencies tailored to the targeted arterial site. More specifically, the transmitting frequency F_t_ is generated in a range between 1.5 and 3.5 MHz for the brachial artery, 4 and 7 MHz for the radial artery, and 7 and 10 MHz for the carotid artery (with an ultrasound sensor with 98% accuracy with a resonant frequency variation of ±5%) to measure the vascular parameters. The range of frequencies is provided to allow for the selection of a specific frequency that is appropriate for the individual subject being examined. The CaNVAS device is tailored for three specific arteries: radial, brachial, and carotid. Each artery is outfitted with its ultrasound sensor and a corresponding algorithm, guaranteeing the emission of acoustic waves at precise frequencies targeted to the specific arterial site.

#### 2.1.2. Pre-Processing Module

The driver circuit is designed in such a way that it guides to transmit the ultrasound acoustic wave at the appropriate frequency towards the target arterial site; the pre-processing module comprises an operational amplifier and a high-pass filter, wherein the received acoustic frequency F_O_ is converted to an electrical signal by a receiver crystal. That electrical signal is amplified by an operational amplifier with a bandwidth greater than 200 MHz and a slew rate of, at a minimum, 4100 Vµs^−1^. The operational amplifier can amplify the electrical signal by a gain value beyond 25. Furthermore, the receiver module is coupled to a high-pass network with a cut-off frequency of 3 MHz to filter the fluctuations on the amplified electrical signal, allowing the sharp amplified electrical signal to microprocessor. This module is programmed with a structural algorithm to extract vascular parameters from the detected reflected acoustic waves. Two checkpoints, Young’s modulus validation to compute the vessel diameter and a diameter-varying function to correct the device position, are included within the algorithm to verify the results obtained simultaneously. The validation unit is also incorporated to validate the pressure value from CaNVAS by the operator. A display module continuously provides the data to the user or attender. The data include the instantaneous systolic blood pressure PS, the instantaneous diastolic blood pressure P_d_, the pulse wave velocity, which comprises vs. and V_d_, the arterial compliance, the stiffness index of the user, the artery position marking, and the device validation report.

#### 2.1.3. Computation of Vascular Parameters

A unique methodology is carried out in CaNVAS to determine the constants α and β, which are fed into the predetermined Equation (1), expressing the nonlinear relationship between the pulse wave velocity and the pressure.

(1) $$P=\alpha V^{2}+\beta \;(kPa)$$

The Doppler shift F_s_, which is computed using the received echo signal from the receiver, is taken as an input for Equation (2) to derive the PWV [37] measure in ms^−1^.

(2) $$PWV=\frac{C\times F_{s}}{2cos\theta \times F_{t}}\;({\mathrm{ms}}^{-1})$$

**Figure 2:**
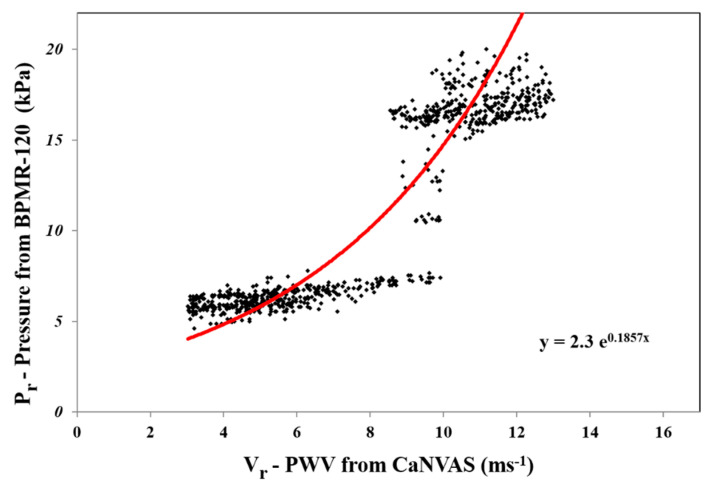
Graph depicts α and β using exponential approximation of Pr and V_r_ data.

C is the speed of the acoustic wave in the artery, which is 1540 ms^−1^; F_S_ is the doppler shift, because the sensors are placed adjacently; and cosθ is taken as the integrated angle value between $cos0^{{}^{\circ}}$ and $cos30^{{}^{\circ}}$, which is 0.5. F_t_ is a transmitter frequency, which is selected in the specific range from about 1.5 MHz to 10 MHz based on the target artery, wherein the target arteries are characterized as the radial artery, the brachial artery, and the carotid artery. The instantaneous pulse wave velocity obtained from the subject is used to extract the peak systolic velocity (V_s_) and the end diastolic velocity (V_d_). The minimum and maximum PWV in one cycle duration is determined, of which 70% of the maximum value is taken as vs. and 10% of it is taken as V_d_. The reference blood pressure (P_r_) parameter from a multitude of 995 random subjects, which included divergent age groups from 21 to 55 (38 ± 17) with various physiological condition (diabetic, high BP, and acute cardiac issues) was measured by means of an external blood pressure measuring device, Diamond Deluxe BPMR-120. Furthermore, the reference pulse wave velocity (V_r_) from the same random subjects using an initial CaNVAS experimental set-up is programmed to compute only the PWV (the procedures were explained to all the subjects included for study and non-objection consent forms were collected before recording the data). These reference pressure values (P_r_) and pulse wave velocity values are plotted on a graph, as depicted in Figure 2, to find the constant values termed as α and β.

**Figure 3:**
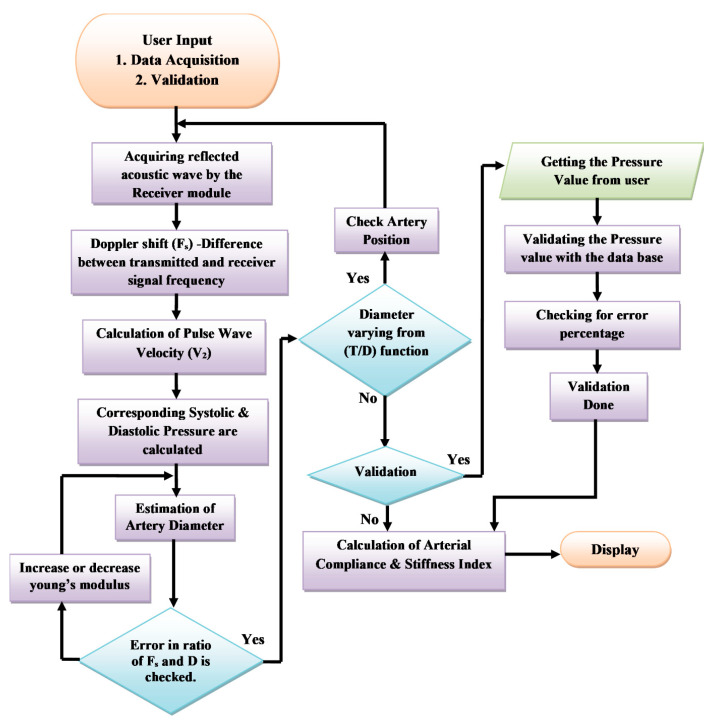
Flowchart representing the process of CaNVAS.

Exponential approximation is employed to analyze the increasing nature of the pressure value to the corresponding increase in the velocity value. From the graph, it is noted that α constant is the slope of the exponential equation, and the y-intercept is calculated when x is zero and the value is taken as β constant. The α constant 0.1857 kPa and the β constant 2.3 kPa values are observed from the curve and set as a standard constant for Equation (1) to calculate the blood pressure value computed from the pulse wave velocity embedded in the non-invasive device CaNVAS. Furthermore, the arterial diameter, arterial compliance, and stiffness index are computed with an appropriate equation, which is embedded in the processor to find the vascular parameters of the user using CaNVAS. Hereafter, the terms “user” and “attendant” refer to the one whose vascular or clinical parameters are specifically measured using the non-invasive device. A sequential algorithm is carried out in the processor, as shown in Figure 3, for the computation of vascular parameters.

Initially, user inputs are received from the choice of either data acquisition or validation. The pre-processed received echoes are fed into the microprocessor and digitized, and the reflected frequency (F_o_) is calculated by a frequency counter as step 1.

(3) $$F_{s}=F_{t}-F_{o}\;(Hz)$$

In step 2, the Doppler shift F_s_ in kilo hertz (KHz) is calculated using Equation (3), where F_s_ will be positive because the blood flows towards the receiver. The pulse wave velocity, which comprises the peak systolic velocity (V_s_) and the end diastolic velocity (V_d_), is computed in step 3 with the same procedure as carried out for finding the reference velocity values using Equation (2). The blood pressure value is derived by applying the velocity values obtained in step 3 and with the derived constants α (0.1857) and β (2.3). The systolic (P_s_) and diastolic blood pressures (P_d_) in kPa are calculated with the corresponding systolic and diastolic velocity values by the two Equations (4) and (5).

(4) $$P_{s}=\alpha V_{s}^{2}+\beta \;(kPa)$$

(5) $$P_{d}=\alpha V_{d}^{2}+\beta \;(kPa)$$

The processor is programmed with a diameter equation to calculate the systolic arterial diameter *D_s_* and the diastolic arterial diameter *D_d_* in millimeters (mm) of the target artery of the user, using Equations (6) and (7).

(6) $$D_{s}=\frac{e^{\left(kP_{s}\right)}t\;E_{o}}{\rho v_{s}^{2}}\;(mm)$$

(7) $$D_{d}=\frac{e^{\left(kP_{d}\right)}t\;E_{o}}{\rho v_{d}^{2}\;}\;(mm)$$

The algorithm used in CaNVAS incorporates various constants. The arterial constant (k) is set to 0.017. The thickness of the artery is predetermined based on the target artery: 0.25mm for the radial artery, 0.29 mm for the brachial artery, and 1.5mm for the carotid artery. The density of blood (ρ) is assigned a value of 1060 kg/m**^3^**. The variables P_s_, P_d_, V_s_, and V_d_ represent the instantaneous systolic and diastolic blood pressures and the velocity values obtained in previous steps. The initial elasticity of the artery (*E_o_*) is set to 13.33 kPa, which will be adjusted according to each subject in subsequent steps. The elasticity property of the artery is determined by its capacity to compress and extend during systole and diastole in one cycle, as reflected in the frequency shift obtained from the receiver. The ratio of the systolic and diastolic diameters is compared to the ratio of the maximum frequency shift to the minimum frequency shift recorded during one cycle. Any differences in these values are considered errors. If the error exceeds a certain threshold, the elasticity value (*E_o_*) is modified from the initial set value, and step 5 is repeated until the error is minimized. Consequently, the final value of *E_o_* is used as the appropriate elasticity value for the user. To ensure system performance, the non-invasive device includes a checkpoint to verify the derived diameter using the diameter equation against the respective arterial site. This derived diameter is correlated with the thickness–diameter function (T/D), allowing for the detection of deviations beyond 0.3 ± 40%. If the deviation of the thickness–diameter value exceeds 40%, it indicates improper device positioning, and this information is displayed on the display module.

If the input received from the user matches the condition described in step 2, the device will execute all the preceding steps exactly as it did for choice 1, including the validation process. Otherwise, it will skip step 8. During the validation phase, only the blood pressure parameter is validated using an external device. This parameter is crucial for calculating the arterial diameter, arterial compliance, and stiffness index, which are the key parameters used to validate the integrated device. The reference blood pressure value obtained from a gold standard BP apparatus, as measured by the user, is compared with the values provided by CaNVAS. The device will display the error value if it exceeds ±0.67 kPa, as is clinically acceptable. Equations (8) and (9) are employed to derive the arterial compliance (AC) in mm^2^kPa**^−^**^1^ and the stiffness index (SI), where ∆P is the difference between the systolic and diastolic blood pressure parameters and ∆D is the change in the systolic and diastolic diameters, calculated in the previous steps using CaNVAS.

(8) $$AC=\frac{\pi \left(D_{s}^{2}-D_{d}^{2}\right)}{4∆P}\;({\mathrm{mm}}^{2}{kPa}^{-1})$$

(9) $$SI=\frac{\ln\left(\frac{P_{s}}{P_{d}}\right)}{\left(\frac{∆D}{D_{d}}\right)}$$

The individual will utilize the user-friendly and non-invasive device CaNVAS to continuously obtain the specified vascular parameter details without any time gaps. A display unit will show the user’s ongoing measurements, including the instantaneous systolic pressure (SYS), instantaneous diastolic pressure (DIA), peak systolic velocity (PSV), end diastolic velocity (EDV), arterial compliance (AC), and stiffness index (SI). Additionally, the device’s validation status will be displayed. The statistical analysis conducted in this study utilized a linear regression model in Microsoft Excel 2016.

## 3. Results

For the performance analysis of CaNVAS, a group of 300 participants was selected. This group consisted of both healthy individuals and individuals with a history of hypertension, hypotension, and cardiovascular disorders. Additionally, subjects undergoing regular renal dialysis were included. The age range of the participants was 21 to 55 years, with an average age of 38 ± 17. Among the participants, 65% were female and 35% were male. This study conducted on an educational campus included a significant number of adolescent students and faculty members as participants. To measure the vascular parameters and blood pressure of these individuals, both CaNVAS and Diamond Deluxe BPMR-120 (Otica Meditronix Co, Vadodara, India.) were used simultaneously. The institute’s ethics committee approved this study, and all participants were provided with a clear explanation of the experimental procedure. Informed consent was obtained from each participant before data acquisition. The typical time required for one set of readings was under two minutes. All subjects underwent comprehensive measurements. In certain cases, a few additional minutes were necessary for the initial device set-up to accurately trace the artery due to the associated challenge. The BPMR-120 cuff was applied to the brachial artery, ensuring it covered at least 80% of the arm’s circumference. CaNVAS was positioned over the target artery (radial artery) using the self-evaluation position correcting unit. During the assessment, all subjects were seated in a relaxed position with their feet resting on the floor and their arms supported at heart level. The measurements were repeated for the same subjects after engaging in physical exercise (Rapid Steps Climbing). Two trials were conducted under each condition to assess the repeatability of CaNVAS. Blood pressure data obtained from CaNVAS were compared to those measured using the gold standard blood pressure device, Diamond Deluxe BPMR-120. Among the total of 600 data points collected for both conditions (normal and after exercise), a few data points were considered outliers due to movement artifacts and powerline interference. These suspected cases of erratic data differences were excluded from the analysis, resulting in a remaining dataset of 556 data points, which accounted for 90% of the total.

### 3.1. Linear Regression Analysis

**Figure 4:**
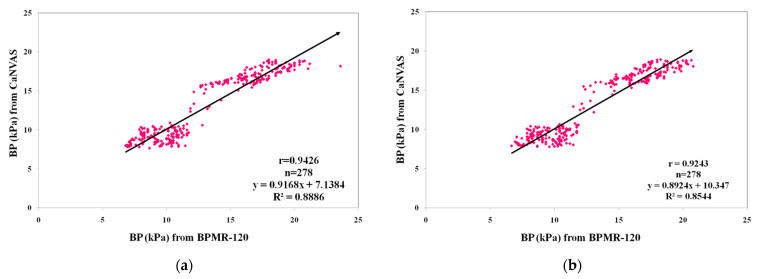
Comparison of blood pressure (BP) measurements from CaNVAS with those obtained from BPMR-120: (**a**) during resting condition; (**b**) after performing physical exercise.

Figure 4 provides an overview of the linear regression analysis conducted on the pressure values obtained from two measurement devices under normal and post-exercise conditions. To ensure compatibility between the devices, a conversion factor (7.501) was applied to convert mmHg to kPa units. The blood pressure results from CaNVAS demonstrated a strong positive correlation with the corresponding values obtained from BPMR-120, which were recorded as whole numbers. Correlation coefficients (r) of 0.94 and 0.92 were achieved for the pressure values measured under normal and post-exercise conditions, respectively. The slightly lower correlation observed after exercise can be attributed to the short time delay involved in setting up the device on the target artery, resulting in sequential measurements rather than simultaneous ones during data recording after exercise.

**Figure 5:**
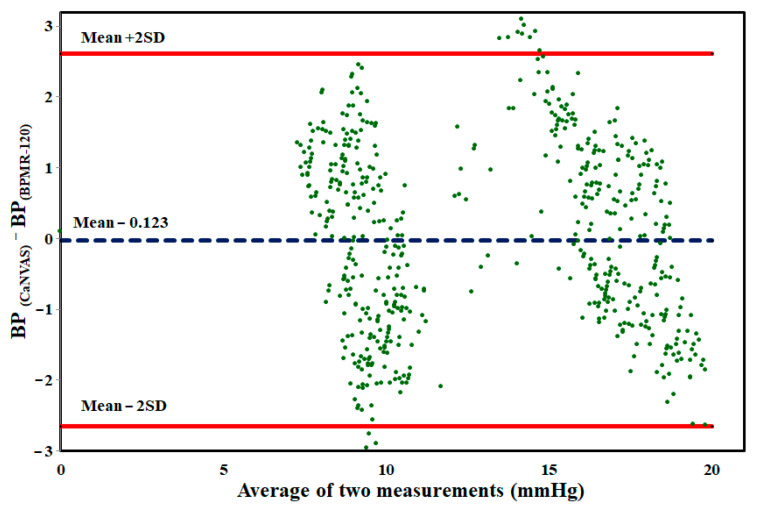
Bland–Altman plot of BP values measured from CaNVAS and BPMR-120 during both resting and after performing physical exercise.

In order to evaluate the agreement between blood pressure values obtained from CaNVAS and the gold standard device under two conditions, Bland–Altman plots were generated with limits of agreement set at ±2 standard deviations (SD), as depicted in Figure 5. The plots reveal a substantial level of agreement between the two measurement devices, with a bias value close to zero. This indicates the reliability of the method employed in CaNVAS for estimating blood pressure based on directly measured pulse wave velocity.

The mean difference between the blood pressure values obtained from the two devices was observed to be −0.123 ± 2.756, indicating minimal distortion in measurements. The graphical representation illustrates that the differences in measurements are randomly distributed, with 98% of the data falling within the range of agreement.

**Figure 6:**
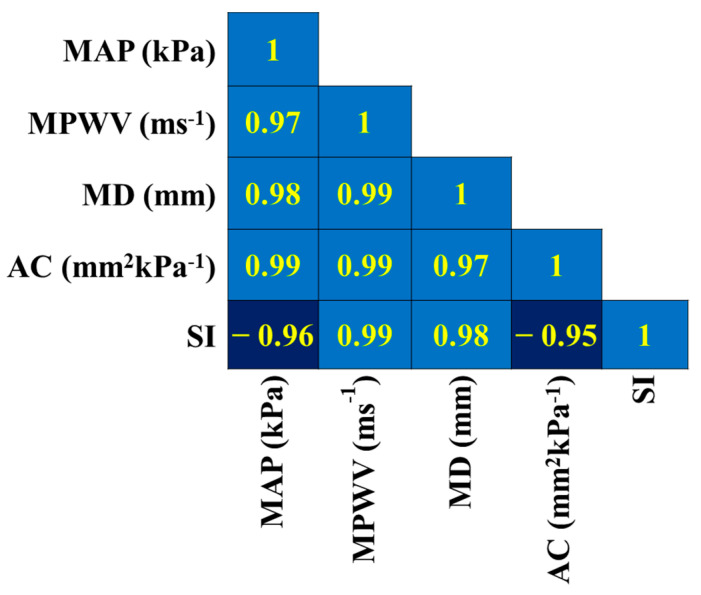
Correlation matrix representing the correlation coefficients calculated between vascular parameters within the subjects.

The correlation matrix is estimated within the vascular parameters (for instance, the Mean Arterial Pressure (MAP), Mean Pulse Wave Velocity (MPWV), Mean Diameter (MD), arterial compliance (AC), and stiffness index (SI)) calculated using CaNVAS for all subjects. The correlation coefficients value depicted in Figure 6 shows a strong relationship between the parameters of the individuals. A positive correlation is shown for the pairs (i) MAP with MPWV, MD, and AC; (ii) MPWV with SI, AC, and MD; and (iii) MD with SI and AC, which implies that a slight increase in one value will be reflected in another, which acts as the biomarker for diagnosing vascular abnormalities. There is a strong negative correlation between the subject’s SI and MAP and AC, which indicates an increase in the Mean Arterial blood pressure value, and arterial compliance decreases the vascular stiffness; this indicates that the forecast estimation methodology carried out in CaNVAS to measure the PWV, BP, AC, and SI is trustworthy.

### 3.2. Repeatability Analysis

**Figure 7:**
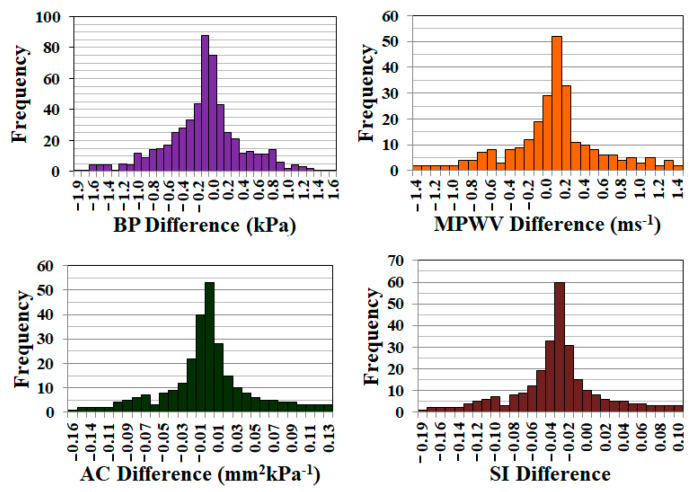
Graph showing the frequency of the error obtained during the measurement of BP, MPWV, AC, and SI in two trials.

During this study, two trials were conducted to assess the repeatability of CaNVAS. The frequency of differences between the two trials for blood pressure (BP), pulse wave velocity (PWV), arterial compliance (AC), and stiffness index (SI) values is depicted in Figure 7. To determine the coefficient of variability (repeatability), the standard deviation of the differences between the two CaNVAS measurements is divided by the average of the mean. The coefficient of repeatability for the parameters is presented in Table 1. This demonstrates the device’s capability to provide accurate evaluations of vascular parameters.

## 4. Discussion

This paper presents a novel device called CaNVAS, along with its methodology, for non-invasively evaluating continuous vascular parameters. CaNVAS utilizes ultrasound technology to measure pulse wave velocity (PWV) based on the reflected frequency shift signal from the artery. This method shows promise and is employed for deriving blood pressure (BP) as well as for estimating arterial compliance (AC) and stiffness index (SI). In a previous device developed by Drzewiecki et al. (1992), an arterial tonometer was used to non-invasively measure continuous blood pressure by identifying the center of the blood vessel [39]. The device required the deflection area of the artery vessel to make contact with the sensor surface for optimal deflection, necessitating a specially designed positioning unit. In contrast, CaNVAS, the proposed device in this study, records ultrasound frequency shifts when placed in proximity to the blood vessel, thereby overcoming the need to involve the entire blood vessel area and the interference angle for observing relevant changes. Another device developed by Jeyaraj et al. (2015) measured arterial compliance and stiffness parameters non-invasively using ultrasound principles [40]. However, this device relied on a one-time measurement of blood pressure using an external device. In comparison, CaNVAS has the advantage of simultaneously measuring instantaneous blood pressure, arterial compliance, and stiffness index, making it a superior solution. Overall, the proposed CaNVAS device offers a novel approach for the non-invasive measurement of continuous vascular parameters, including blood pressure, arterial compliance, and stiffness index, utilizing ultrasound technology and overcoming the limitations of existing devices.

This paper provides a comprehensive description of the hardware and algorithm of the CaNVAS device. The performance of the device was assessed using approximately 300 subjects from various age groups, ranging between 18 and 35 years old, with an average age of 26 ± 9. The blood pressure values measured by CaNVAS were compared to those obtained from a sphygmomanometer (BPMR-120), demonstrating an accuracy rate of 95%. The reliability of other parameters, such as arterial compliance (AC) and stiffness index (SI), measured by CaNVAS was evaluated using a self-correlation technique. Because these parameters are interconnected with blood pressure, even a slight error in blood pressure measurement can impact AC and SI values. It was observed that AC and SI measured by CaNVAS are influenced by age. Similar to the findings of Jayaraj et al. in their ARTSENS device, there is a gradual decrease in arterial compliance and an increase in stiffness index with advancing age.

To ensure the repeatability of CaNVAS, linear regression analysis and Bland–Altman plots were employed. The results demonstrated an average coefficient of variability of 12.5%, which is consistent with previous research and indicates a comparable level of repeatability.

## 5. Conclusions

The obtained results provide strong evidence that CaNVAS is a promising device for measuring vascular parameters comparable to existing modalities. Its compact size, affordability, repeatability, and user-friendly nature make CaNVAS suitable for medical applications, such as measuring continuous blood pressure during dialysis procedures and monitoring the health status of subjects after cardiac surgery. However, it is important to note the limitations of this study. The hysteresis behavior of psychobiological variables during exercise was not considered, and this aspect will be investigated in future research. Additionally, the study population mainly consisted of adolescent students and middle-aged faculty members due to its location on an educational campus. This demographic bias represents a limitation, and a demographic analysis of CaNVAS will be explored in future studies.

## Figures and Tables

**Table 1:** Coefficient of repeatability for CaNVAS measurements.

Parameters Measured	Coefficient of Repeatability in %
Blood Pressure (kPa)	10.2
Pulse Wave Velocity (ms^−1^)	11.3
Arterial Compliance (mm^2^kPa^−1^)	20.9
Stiffness Index	7.6

## Data Availability

The clinical data are available upon request in accordance with the volunteers’ informed consent. The data will not be shared online.
